# Psychological Interventions for Internalized Weight Stigma: A Systematic Review of Feasibility, Acceptability, and Preliminary Efficacy

**DOI:** 10.21203/rs.3.rs-4844880/v1

**Published:** 2024-08-29

**Authors:** Laura D’Adamo, Abigail T. Shonrock, Lawrence Monocello, Jake Goldberg, Lauren H. Yaeger, Hiba Jebeile, Rebecca Pearl, Denise E. Wilfley

**Affiliations:** Drexel University; University of Florida College of Public Health and Health Professions; Washington University in St Louis School of Medicine; Washington University in St Louis School of Medicine; Washington University School of Medicine; The University of Sydney; University of Florida College of Public Health and Health Professions; Washington University in St Louis School of Medicine

**Keywords:** internalized weight stigma, weight bias, body image, psychological intervention, weight

## Abstract

**Background:**

Internalized weight stigma (IWS) is highly prevalent and associated with deleterious mental and physical health outcomes. Initiatives are needed to address IWS and promote effective coping and resilience among individuals who are exposed to weight stigma. We conducted a systematic review of psychological interventions for IWS and examined their feasibility, acceptability, and preliminary efficacy at reducing IWS and related negative physiological and psychological health outcomes.

**Methods:**

Eight databases were searched. Inclusion criteria included: (1) psychological intervention; (2) published in English; and (3) included internalized weight stigma as an outcome. Exclusion criteria included: (1) commentary or review; and (2) not a psychological intervention. A systematic narrative review framework was used to synthesize results.

**Results:**

Of 161 articles screened, 20 were included. Included interventions demonstrated high feasibility, acceptability, and engagement overall. Sixteen of 20 included studies observed significant reductions in IWS that were maintained over follow-up periods, yet data on whether interventions produced greater reductions than control conditions were mixed. Studies observed significant improvements in numerous physical and mental health outcomes.

**Conclusions:**

Findings indicate that existing interventions are feasible, acceptable, and may provide meaningful improvements in IWS and associated health outcomes, highlighting the potential for psychological interventions to promote improved health and wellbeing in individuals with IWS. Additional research using rigorous study designs (e.g., randomized controlled trials) is needed to further evaluate the efficacy of interventions for IWS.

## Plain English Summary

This study conducted a systematic review of psychological interventions for internalized weight stigma (IWS). We examined the feasibility, acceptability, and preliminary efficacy of existing interventions at reducing IWS and related negative physiological and psychological health outcomes. Results indicated that existing psychological interventions are feasible, acceptable, and may provide meaningful improvements in IWS and associated health outcomes over long-term periods. Findings support the potential for psychological interventions to promote improved health and wellbeing in individuals with IWS.

## Background

Decades of research have documented that weight stigma (i.e., the societal devaluation and mistreatment of individuals that results from negative attitudes, beliefs, and stereotypes based on weight) is a global public health concern (1–3). Experiences of weight stigma and discrimination, which are widespread in employment and health care settings (4–7), are robustly associated with a myriad of adverse physiological outcomes (e.g., increased diabetes risk, dysregulated cortisol, oxidative stress) and mental health issues (e.g., depression, disordered eating, low self-esteem) (8, 9). Longitudinal research has also implicated weight stigma as a contributing factor to the maintenance of obesity and related diseases (10, 11). Individuals often internalize the pervasive negative stereotypes based on weight (e.g., that individuals with higher weight are lazy or lack willpower), resulting in internalized weight stigma (IWS) (12, 13), also referred to as “weight self-stigma” or “weight bias internalization.” An estimated 40–50% of U.S. adults with higher weight have IWS (14). IWS is associated with negative mental and physical health outcomes, including depression, chronic stress, and disordered eating (15–18) and has been linked to healthcare avoidance (6, 19). Initiatives are needed to address IWS and its deleterious effects on physical and mental health and to promote coping and resilience among individuals who experience weight stigma.

Although public health campaigns and policies to reduce weight stigma have been introduced and evaluated (20, 21), strategies for addressing IWS have received less attention. Research on strategies for reducing mental health self-stigma suggests that psychological interventions aimed at reducing IWS represent a promising approach for changing individuals’ self-stigmatizing beliefs, increasing self-esteem and empowerment, and promoting effective coping (22). In recent years, numerous studies developing and evaluating novel psychological interventions to address IWS have aimed to elucidate the potential for these interventions to reduce IWS and associated mental and physical health outcomes. It has also been proposed that intervention components targeting IWS may be integrated into lifestyle modification interventions to improve individuals’ ability to engage in behavioral lifestyle changes, given that IWS is associated with shame and poor self-efficacy, which may interfere with these abilities (23–25).

No study to date has reviewed psychological interventions for IWS and associated health outcomes. As such, we conducted a systematic review to synthesize studies examining components of existing interventions, their feasibility and acceptability, and their preliminary efficacy at reducing IWS and related negative physiological and psychological health outcomes. As research in this area is limited, we included interventions specifically designed to target IWS, including adjunctive IWS interventions integrated within other interventions, as well as interventions that did not explicitly target IWS but included it as an outcome.

## Methods

This systematic review was conducted in accordance with the Preferred Reporting Items for Systematic Reviews and Meta-Analyses (PRISMA) guidelines (26).

### Literature Search and Study Selection

A medical librarian (LHY) searched the literature for records including the concepts of internalized stigma, body weight, cognitive-behavioral therapy, self-compassion therapy, and psychological interventions. The librarian created search strategies using a combination of keywords and controlled vocabulary in Embase.com 1947-, Ovid Medline 1946-, Scopus 1823-, Cochrane Central Register of Controlled Trials (CENTRAL), The Cochrane Database of Systematic Reviews (CDSR), Cumulated Index to Nursing and Allied Health Literature (CINAHL Plus) 1937-, APA PsycInfo 1800s-, and Clinicaltrials.gov 1997-.

All search strategies were completed 3/26/2024 with no added limits and a total of 326 results were found. Duplicate records (n = 148) were deleted using Covidence.org resulting in a total of 178 unique citations included in the project library. Fully reproducible search strategies for each database can be found in the **Appendix**.

### Inclusion and Exclusion Criteria

Identified articles were screened based on the following inclusion criteria: (1) Psychological intervention (e.g., acceptance and commitment therapy, cognitive-behavioral therapy, self-compassion therapy); (2) Published in English; and (3) Included a measure of internalized weight stigma or bias as an intervention outcome.

Identified studies were excluded based on the following exclusion criteria: (1) Commentary or review paper; (2) Not a psychological intervention (e.g., public health campaigns to reduce weight stigma); and (3) Interventions aimed to reduce weight stigmatizing attitudes in healthcare professionals.

### Data Extraction and Synthesis

Identified articles were uploaded into Covidence systematic review software. Article titles and abstracts were independently screened for relevance by the first four authors. Two authors each conducted full text reviews and consulted with one another to resolve conflicts.

The following information was extracted from the articles: year of publication, country, study design, sample characteristics (e.g., sample size, demographics, weight, baseline IWS), type of psychological intervention evaluated, and data on the primary outcome of interest (i.e., IWS) and other relevant psychosocial or physiological health outcomes assessed (e.g., body image, weight, disordered eating behaviors, self-compassion, depression) following the intervention.

## Results

### Article Selection

**Figure 1:**
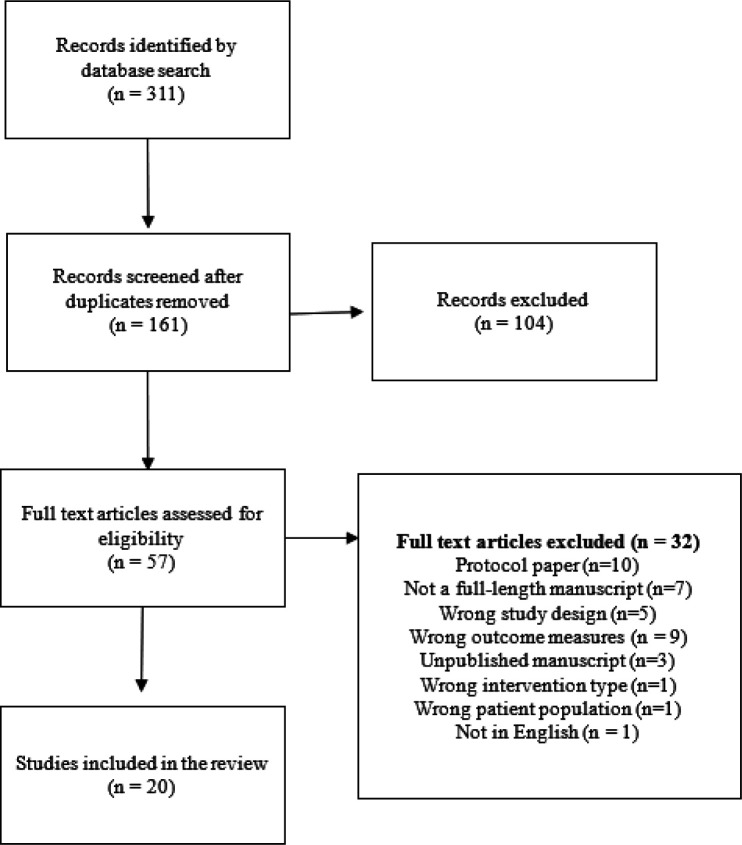
Flow chart of study search and selection.

A total of 161 articles identified in the search following the removal of duplicates were screened, of which 106 were excluded from full-text review. Of the 57 full texts assessed for eligibility, 32 were excluded for the following reasons: Protocol paper (n = 10), not a full-length manuscript (n = 7), wrong study design (n = 5), wrong outcome measures (n = 7), unpublished manuscript (n = 3), wrong patient population (n = 1), wrong intervention type (n = 1), and not in English (n = 1). Thus, 20 articles met eligibility criteria and were included in the review. See Figure 1 for a PRISMA flow chart of the article search and selection process.

### Study Characteristics

Study characteristics for the 20 articles included in this review are presented in **Table 1**. Included studies were conducted between 2010 and 2023 and were most often conducted in the USA (n = 16) (23,25,27–41), followed by Portugal (n = 2) (42,43), Australia (n = 1) (44), and Canada (n = 1) (37). Study designs included pilot trials (n = 10) (23,27–30,33,34,36,43,44), randomized controlled trials (RCTs) (n=9) (25,31,35,37–42), and one proof-of-concept trial (32). The most common intervention approaches were acceptance and commitment therapy (n = 5) (34,35,40,42,43), followed by cognitive-behavioral therapy (n = 3) (23,38,39), self-compassion interventions (n = 3) (28,33,44), weight neutral or intuitive eating interventions (n = 2) (29,36), behavioral interventions (n = 2) (25,30), yoga interventions (n = 2) (27,41), one positive psychology and motivational interviewing intervention (32), one journaling intervention (31), and one implicit stereotype retraining intervention (37). Nine studies evaluated lifestyle modification interventions (i.e., interventions targeting dietary or physical activity change or weight management) (25,28,30,32,35,38,39,42,43), 2 of which evaluated the addition of cognitive-behavioral intervention modules targeting IWS to behavioral weight loss treatment (38,39) and one of which evaluated the addition of a mindful self-compassion intervention following behavioral weight loss treatment (28); 8 studies evaluated interventions focused on weight stigma, IWS, or body gratitude (23,31,33,34,37,40,41,44); 2 studies evaluated interventions targeting disordered eating (29,36); and one study evaluated a stress management intervention (27). Intervention duration ranged from 3 weeks (33) to 72 weeks (20-weeks of group treatment followed by 52 weeks of monthly and every-other-month sessions) (39). Formats included group sessions (n = 13) (23,25,27–30,33,35,38,39,42–44); online courses (n = 2) (36,37); telephone-based interventions (n = 1) (32); and an individual writing-based intervention (n = 1) (31).

The sample sizes of included studies ranged from 12 to 162 participants. Most studies (n = 11) recruited mixed gender samples (23,25,27,30,32,34–36,38–40), and 9 studies enrolled only women (28,29,31,33,37,41–44). The mean age of participants spanned from 20.1 (29) to 53.4 years (23). Three studies (29,31,41) recruited college-aged participants. The racial composition of the samples varied; most studies predominantly enrolled White participants, whereas 3 studies (23,29,38) recruited samples that were <50% White. Studies most commonly targeted participants with body mass indexes (BMIs) above or equal to 25 (n = 10) (27,28,30,33–35,41–44), 5 studies targeted participants with BMIs above or equal to 30 (23,25,28,38,39), one study targeted participants with BMIs above or equal to 27.5 (40), and one study targeted participants who self-identified as living with obesity (37). Eight studies specifically targeted participants with heightened IWS (23,28,31,33,34,38–40).

### Measurement of IWS

Measurement of IWS was highly consistent across included studies, with 8 studies (23,28,30,33,38,39,41,44) using the Weight Bias Internalization Scale (WBIS) (12); 9 studies (25,28,34,35,38–40,42,43) using the Weight Self-Stigma Questionnaire (WSSQ) (45); and 6 studies (27,29,31,32,36,37) using the Modified Weight Bias Internalization Scale (WBIS-M) (46), a modified version of the WBIS for individuals of all body weights. These measures were used to evaluate changes in IWS from baseline to post-intervention and over follow-up periods. The WBIS was developed to assess the degree to which respondents believes that negative stereotypes about individuals with higher weight apply to them and includes items such as: “As an overweight person, I feel that I am just as competent as anyone”; “I am less attractive than most other people because of my weight”; and “I hate myself for being overweight” (12). Some in the WBIS-M were modified to be applicable to individuals who do not have higher weight, such as: “Because of my weight, I feel that I am just as competent as anyone” and “I hate myself for my weight” (46). The WSSQ was developed to assess self-devaluation based on weight and fear of enacted weight stigma; it includes items such as: “I’ll always go back to being overweight”; “I feel guilty because of my weight problems”; and “People think that I am to blame for my weight problems” (45).

### Feasibility and Acceptability of Interventions

Of the 20 included studies, 13 reported on feasibility, acceptability, and engagement metrics (23,28,29,32–35,38–42,44). Most studies (n = 8) reported high acceptability ratings, with participants finding the interventions relevant and useful (23,34,35,38–42). Pearl et al. (39) found higher acceptability and greater change attitudes among participants in the intervention group (behavioral weight loss plus a cognitive-behavioral intervention for IWS) relative to the control group (behavioral weight loss alone). Session attendance was generally high across studies; one study with two arms reported higher attendance and adherence rates in intervention conditions compared to controls (29), whereas another study found lower attendance and engagement in the intervention group relative to control (28).

### Effects of Psychological Interventions on IWS

The majority of the included studies (n = 16) reported significant reductions in IWS from baseline to post-intervention and at follow-up assessments (23,28–32,34–36,38–44). Among studies with control conditions, 6 studies found greater decreases in IWS in the intervention group relative to controls (23,31,35,38,39,42), whereas 5 found no differences between conditions (28–30,40,41). While both the WBIS and WSSQ measures were represented in studies which did and did not report differences between conditions, Pearl et al.’s studies (38,39) found that changes in WBIS scores did not differ between intervention and active control groups, whereas decreases in WSSQ scores were greater in the intervention condition. Observed decreases in IWS measures were sustained over long-term follow-ups in most studies that detected effects, with follow-up periods ranging from one-week (31) to 72-weeks (39).

### Other Psychosocial and Physiological Outcomes

In addition to reductions in IWS, several studies (n = 19) reported improvements in a range of psychosocial outcomes, including internalized shame, self-compassion, disordered eating, intuitive eating, quality of life, body dissatisfaction, and body appreciation, yet the degree to which outcomes improved more in intervention vs. control conditions were mixed (23,25,27–36,38–44).

Physiological outcomes, such as improvements in physical activity, blood pressure, and HDL cholesterol were observed in numerous studies (n = 9); data were mixed regarding whether outcomes improved more in intervention vs. control conditions (25,28,32,35,38–40,42,43). Reductions in weight were common among studies evaluating IWS interventions in combination with lifestyle modification programs (n = 7) (25,28,30,38,39,42,43).

## Discussion

This review synthesized findings from 20 studies evaluating the feasibility, acceptability, and preliminary efficacy of psychological interventions for IWS and their impact on related health outcomes. Table 2 outlines key terms and findings.

Of the 20 included studies, 65% reported on feasibility, acceptability, and engagement metrics. Most of these studies demonstrated high feasibility, acceptability, and session attendance. Most studies (n = 16) reported significant reductions in IWS from baseline to post-intervention and at follow-up assessments. These reductions were observed across numerous intervention types including behavioral weight loss, body gratitude journaling, physical activity promotion, and weight stigma interventions and across modalities including group formats, guided self-help, and online courses. However, data on whether interventions produced greater reductions than control conditions were mixed. Six studies with control conditions showed greater decreases in IWS in the intervention groups compared to controls, whereas 5 found no differences (i.e., the intervention and control groups experienced equal reductions in IWS). This finding may suggest that some interventions are more efficacious at addressing IWS, or that common factors within the interventions and control conditions (e.g., supportive group treatments) were helpful for reducing IWS. Of note, both the WBIS and WSSQ were represented in studies which did and did not report differences between conditions. However, Pearl et al. (38, 39) found that changes in WBIS scores did not differ between intervention (behavioral weight loss plus a cognitive-behavioral intervention for IWS) and active control (behavioral weight loss alone) groups, whereas decreases in WSSQ scores were greater in the intervention condition. This finding may suggest that there are meaningful differences in measurement of IWS constructs between the WBIS and WSSQ. For example, although both measures assess self-devaluation based on weight, the WSSQ also captures perceived weight stigma enacted by others (e.g., “People discriminate against me because I’ve had weight problems”) (45); this construct may have been more sensitive to intervention effects in the above studies. Another possible explanation for the discrepancy in findings is that participants in these two studies were selected based on a WBIS cut-off score (but not a WSSQ cut-off score), which may have resulted in less variability in WBIS scores in the sample.

In addition to reductions in IWS, several included studies reported improvements in a range of psychosocial and physiological outcomes, such as increased physical activity, improved blood pressure, and HDL cholesterol levels. Studies that included IWS interventions in combination with lifestyle modification focused on weight management were particularly effective in producing weight loss. These findings highlight the potential for interventions to significantly improve both IWS’s mental health correlates and associated negative clinical outcomes. While lifestyle modification programs with added IWS intervention components did not produce greater weight losses than lifestyle modification alone (38, 39), a finding which does not support the hypothesis that addressing IWS may further improve weight loss (25), neither do IWS interventions negatively impact weight outcomes. IWS interventions may be an important addition to lifestyle modification programs to prevent the emergence of, or reduce existing, IWS in patients.

This review served as an important first step to demonstrate that psychological interventions can produce meaningful reductions in IWS, a critical direction given the harmful effects of IWS on physical health, mental health, and healthcare services use (15, 17). Strengths of the review included the rigorous methodological approach used for study selection, which included the involvement of a medical librarian and screening of over 150 studies. Limitations included that many included studies had small, homogeneous samples. Further research in larger, more diverse samples is needed. Further, the variability in types of interventions evaluated (e.g., lifestyle modification, body appreciation interventions) and psychological approaches used (e.g., self-compassion, mindfulness and acceptance) preclude us from being able to identify intervention components or approaches that effectively reduce IWS. Future research is needed to rigorously evaluate interventions using RCTs and examine the mechanisms through which these interventions impact IWS and related health outcomes.

## Conclusions

This review synthesized the existing literature on psychological interventions for the reduction of IWS. Findings indicated that existing interventions are feasible, acceptable, and may provide meaningful improvements in IWS and associated health outcomes, highlighting the potential for psychological interventions to promote improved health and wellbeing in individuals with IWS.

## Figures and Tables

**Table 2:** Key terms and findings.

Key Terms	
Weight stigma	Societal devaluation and mistreatment of individuals that results from negative attitudes, beliefs, and stereotypes based on weight
Internalized weight stigma	An individual’s acknowledgement and agreement with negative societal weight-based beliefs and attitudes
Key Findings	
• Research on psychological interventions targeting internalized weight stigma has dramatically increased in recent years. • Interventions using psychological approaches to promote body acceptance, self-compassion, and reject weight stigma demonstrate feasibility and acceptability. • Data on whether interventions produced greater reductions than control conditions were mixed. • Additional research using rigorous study designs (e.g., randomized controlled trials) is needed to further evaluate the efficacy of interventions for IWS.	

## Abbreviations

BMI: body mass index
IWS: internalized weight stigma
WBIS: Weight Bias Internalization Scale
WBIS-M: Weight Bias Internalization Scale-Modified
WSSQ: Weight Self-Stigma Questionnaire
